# Trends in Median Age at First Sex and Age at First Marriage Among Youth in Tanzania: Accelerated Failure Time Model (1994–2016)

**DOI:** 10.1007/s10508-025-03282-4

**Published:** 2026-01-16

**Authors:** Jacqueline Materu, Eveline T. Konje, Ties Boerma, Mark Urassa, Milly Marston, Emma Slaymaker, Jim Todd

**Affiliations:** 1 Sexual and Reproductive Health Program, National Institute for Medical Research, Mwanza Centre, P.O Box 1462, Mwanza, Tanzania; 2 Department of Biostatistics, Epidemiology and Behavioural Sciences, School of Public Health, Catholic University of Health, and Allied Sciences, Mwanza, Tanzania; 3 Institute for Global Public Health, University of Manitoba, Winnipeg, MB Canada; 4 Department of Population Health, London School of Hygiene and Tropical Medicine, London, UK

**Keywords:** Age at first sex, Age at first marriage, Survival analysis, Accelerated failure time model, Youth

## Abstract

Understanding trends in age of first sex and first marriage is vital for interventions addressing sexually transmitted infections (STIs/HIV) and youth sexual behavior. Shifts in these milestones affect fertility, contraceptive use, and STI/HIV dynamics. Traditional descriptive statistics often overlook younger populations, leading to inaccurate trend assessments. This study analyzed trends in median age at first sex and first marriage using survival analysis. Data from eight surveys within Magu Health and Demographic Surveillance System (1994–2016) were analyzed, focusing on individuals aged 15–24 years. The accelerated failure time (AFT) model with log-logistic distribution estimated these medians. Results showed an increase in median age at first sex by one year for both sexes and in first marriage by one year for females and two years for males. The AFT model captured gradual increases from 2003–2004 to 2015–2016 for females and 2003–2004 to 2010 for males, while standard descriptive statistics showed no changes in specific periods: age at first sex, 1996–1997 to 2012–2013 (females) and 2003–2004 to 2012–2013 (males); first marriage, 1994–1995 to 2015–2016 (females) and 2003–2004 to 2010 (males). Individuals with no education had lower median age at first sex (males: 17.1 vs. 18.3 years; females: 16.2 vs. 18.2) and first marriage (females: 18.0 vs. 21.3) than those with secondary or higher education. HIV-positive individuals experienced slightly earlier age at first sex and first marriage than negative individuals. Education plays a pivotal role in delaying these events. The AFT model enriches trend assessment.

**Supplementary Information:**

The online version contains supplementary material available at 10.1007/s10508-025-03282-4.

## Introduction

Evaluating trends in the age of first sexual intercourse and marriage is crucial for designing and evaluating effective programs targeting sexually transmitted infections (STIs/HIV) and adolescent sexual behavior (Adair, 2008; Onsomu et al., 2013; Shrestha et al., 2016). Typically, these milestones occur in the later teenage years (Gupta & Mahy, 2003; Ryu, 2023; SPOUSES, 2001; Wellings et al., 2006), and even slight shifts in the median age, by just a year or two, are considered epidemiologically significant. Such shifts can influence fertility rates, contraceptive use, and the length of the period of premarital sex (Magadi & Agwanda, 2010; Marston et al., 2017; Wellings et al., 2006). Changes in the period between age at first sex and age at first marriage had significant implications for the HIV epidemic, as a longer period of premarital sex increases the exposure risk to HIV and other STIs (Ajaegbu, 2015; Wellings et al., 2006). Additionally, studies have shown that age at first sex is associated with rates of contraceptive use, which can affect population growth and the burden on healthcare systems (Fagbamigbe, 2021; Magnusson et al., 2012).

Trends in the timing of age at first sex and age first marriage have been extensively studied, as they play a crucial role in understanding family formation, gender dynamics, and risky behaviors related to sexual and reproductive health (Cohen & Bessinger, 2003; Mensch et al., 2006; Wellings et al., 2006; Zaba et al., 2004). However, the estimating trends from surveys is challenging, due to inconsistencies in survey questions, analysis methods, and the quality of self-reported data (Materu et al., 2023; Mensch et al., 2003, 2014; Wringe et al., 2009; Zaba et al., 2004). Some studies address inconsistencies and estimate the median (Upchurch et al., 2002; Wringe et al., 2009), while others estimate without correction (Materu et al., 2023). Other studies have observed different approaches, including the implementation of various data collection techniques to enhance privacy and consistency, with little to no improvement found (Mensch et al., 2003, 2014).

The major source of age at first sex and age first marriage information remains self-reported data collected through nationally representative household surveys conducted in many countries (Ministry of Health et al., 2016; Ministry of Health (MoH) [Tanzania Mainland] et al., 2022; National Bureau of Statistics & ICF Macro, 2011). Estimating changes in trends after correcting for inconsistencies using robust statistical methods helps increase confidence in these sensitive self-reported data, especially for the young people, and informs interventions and policy decisions.

The reported ages have often been used to estimate the mean or median age at first sexual intercourse and marriage using a standard descriptive statistics approach (traditional method), typically focusing only on individuals who have already experienced these events, which often leads to the exclusion of those who have not (Becker et al., 2018; Nahar et al., 2013). This method often emphasizes older age groups where these milestones are more universally experienced. In contrast, a survival analysis approach includes censored observations, allowing for the consideration of all individuals, including younger age groups (15–19 and 20–24). In Tanzania, data on age at first sex and age at first marriage are typically gathered through national surveys like the Demographic and Health Survey and Malaria Indicator Survey (TDHS-MIS) (Ministry of Health (MoH) [Tanzania Mainland] et al., 2022). However, the estimates from the standard descriptive statistics for estimating the median age at first sex and age at first marriage for age groups 15–24 or 15–19 years are often unavailable because fewer than half of the respondents have experienced these events. This means the reported estimations, for older individuals, may be outdated, and not applicable to current adolescents, who may have different experiences. In contrast to the standard descriptive statistics, which require more than 50% of respondents to have experienced the event, survival analysis includes all data, including censored cases who have not yet experienced the event, to estimate median age at first sex and age at first marriage (Ministry of Health (MoH) [Tanzania Mainland] et al., 2022; Zaba et al., 2002). Using survival analysis, such as accelerated failure time (AFT) models, allows inclusion of all individuals, even those who have not yet experienced these events, to estimate median ages more accurately. Consequently, survival analysis can yield more timely and relevant estimates compared to conventional descriptive statistics (Zaba et al., 2002).

In survival analysis, the Kaplan–Meier and Life Table methods have been used to estimate the median age at first sex and age at first marriage (Cremin et al., 2009; Zaba et al., 2002). These two techniques are both nonparametric methods, meaning they do not rely on assumptions about the underlying distribution of the data. Instead, they utilize the observed data directly to make estimations, making them flexible and robust approaches for analyzing survival data. Previous studies have shown that accelerated failure time (AFT) models with a log-logistic distribution fit the data well and are more useful for estimating or predicting the median ages of the events (Eaton, 2022; Materu et al., 2023).

The AFT model directly analyzes time to event, assuming a multiplicative effect of covariates on survival time, unlike proportional hazard (PH) models that focus on the survival function (Swindell, 2009). While less commonly used than Cox PH models for analyzing age at first sex and age at first marriage, the AFT model is highly adaptable and versatile. A prior study using the same dataset as this one found that the AFT model with a log-logistic distribution outperformed other survival models in analyzing these events across survey rounds (Materu et al., 2023). Its flexibility allows for various hazard shapes (e.g., increasing, decreasing, or constant), accommodates time-varying covariates, and facilitates robust prediction and inference (Saikia & Barman, 2017).

The current study aimed to evaluate the trends in age at first sex and age at first marriage among the young population aged 15–24 years in Magu-Kisesa, Tanzania, using the accelerated failure time (AFT) model with a log-logistic distribution for corrected inconsistency data. The study estimates the median age at first sex and age at first marriage, overall and stratified by sex, education level, and HIV status, to show trends over 22 years from 1994 to 2016.

## Method

### Participants

We utilized data from eight serological surveys conducted at an average interval of three years within the Magu Health and Demographic Surveillance System (HDSS), also known as the Kisesa Observational HIV Cohort Study, from 1994 to 2016. Magu HDSS is an open community cohort located 20 km east of Mwanza city. Detailed descriptions of the sampling strategies and survey methods employed in Magu HDSS can be found elsewhere (Urassa et al., 2024). The analysis focused on individuals aged 15–24 years who attended at least one survey between 1994 and 2016. Males and females were analyzed separately for all participants aged 15–24 years living in the cohort each year between 1994 and 2016.

### Measures

The surveys collected information on age at first sexual intercourse and age at first marriage through two questions: whether the respondent had engaged in sexual activity or had been married, and if so, at what age (in completed years) they first experienced the event. Additionally, data were collected on the respondent’s sex, education level, age, date of birth, interview date, residence area, and other survey parameters (not relevant for the current analysis). It is important to note that these surveys did not distinguish between formal marriage and cohabitation (living with a partner), which may influence reported ages at first marriage.

Round 3 (1999–2000) lacked data on age at first sex, and rounds 2 (1996–1997) and 3 lacked data on age at first marriage; however, all rounds provided information on whether participants had experienced sex or marriage, so we did not exclude these years from analysis (Materu et al., 2025). From these data (over the reported serological surveys), we compiled the “best” estimate of age at first sex and age at first marriage corrected for inconsistencies (Materu et al., 2025).

### Procedure

Trends over time in age at first sex and first marriage were analyzed by determining the median age for these events between 1994 and 2016 among those aged 15–24 years. We also compared estimates from the Magu HDSS cohort with those from the Tanzania Demographic and Health Survey (TDHS), focusing on young adults (aged 20–24 years) as reported by TDHS for that age group and for adults aged 25–49 years (ICF, 2012).

### Statistical Analysis

We used a parametric survival method (accelerated failure time model [AFT] with a log-logistic distribution) to calculate the median age at first sex and age at first marriage. The AFT model was used as it was identified as the best model in a previous study using similar data (Eaton, 2022; Materu et al., 2023). We also used traditional descriptive statistics to calculate the median from the reported age at first and age at first marriage, and compared the estimates from the AFT results. The calculation of the median from the standard descriptive statistics includes those who experienced the events (i.e., it does not account for censoring).

The survival curves showing the estimated probability of first sex and marriage experience using the log-logistic AFT model were generated. All analyses were performed using STATA version 18 and R version 4.3.0.

## Results

### Trend in Age at First Sex

**Fig. 1:**
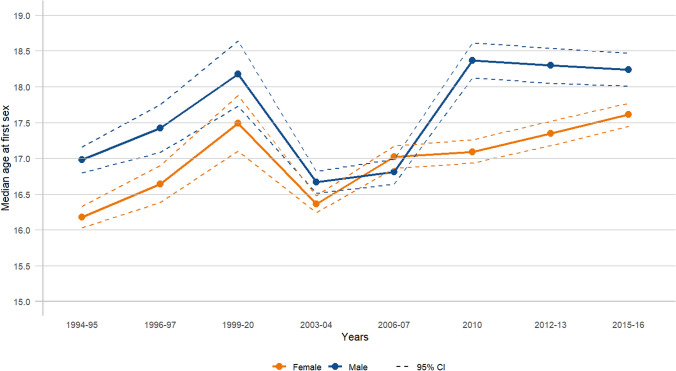
Trends in median age at first sex by survey year, females and males (15–24 years)

**Fig. 2:**
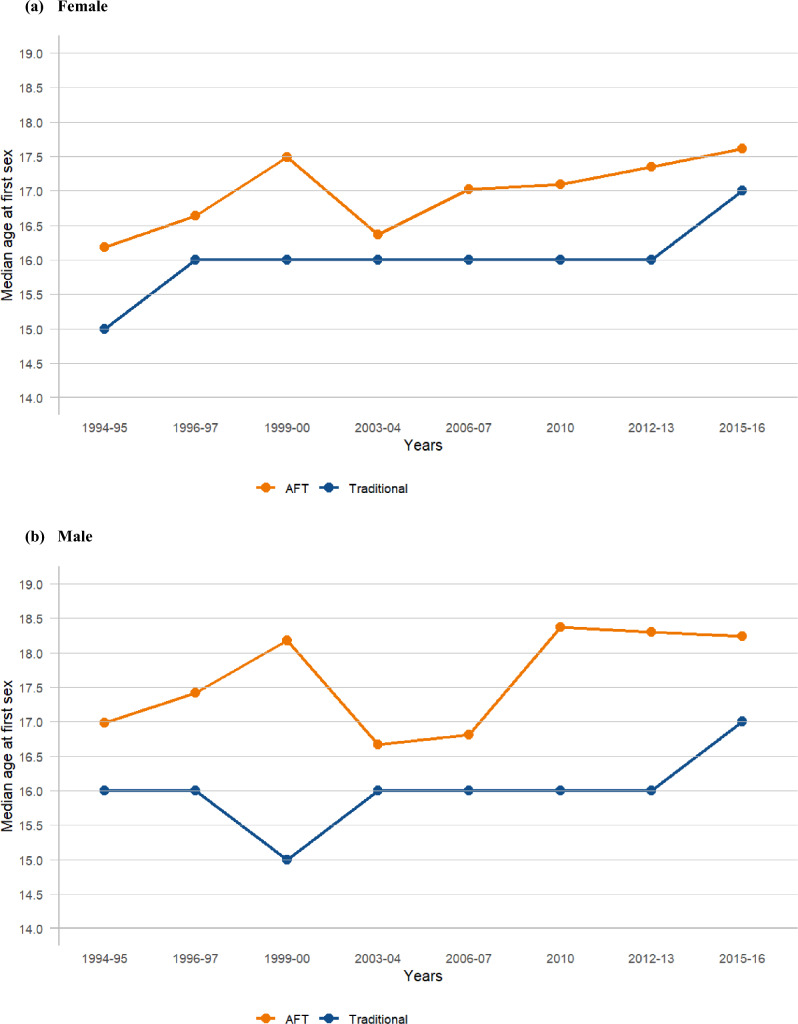
Trends in median age at first sex estimated using AFT and traditional methods (15–24 years)

Figure 1 and Appendix: Table 1 show the trend in the estimated median age at first sex from the accelerated failure time (AFT) model for both sexes. Overall, the median age at first sex increased by one year for both females (from 16.18 years in 1994–1995 to 17.61 years in 2015–2016) and males (from 16.98 years in 1994–1995 to 18.24 years in 2015–2016), with the median age at first sex for males consistently higher than for females. A slight systematic increase was observed for females, with the median age at first sex rising from 16.36 years in 2003–2004 to 17.61 years in 2015–2016, while males experienced a slight decline in the median age at first sex from 18.37 years in 2010 to 18.24 years in 2015–2016. For both sexes, the median age at first sex initially increased from 1994–1995 to 1999–2000 (females: 16.18–17.49; males: 16.98–18.18), followed by a drop and then a subsequent increase. When comparing the trends in the estimated median age at first sex using the AFT model (a survival method) with those obtained using the traditional method (median from descriptive statistics), the overall median age at first sex was higher with the AFT model for both females and males. Additionally, the AFT model was observed to capture small changes more effectively than the traditional method, which showed no changes (constant or flat trend) in some years for both females and males (females: 16.00–16.00 from 1996–1997 to 2012–2013; males: 16.00–16.00 from 2003–2004 to 2012–2013) (Fig. 2a and b) (Appendix: Table 1).

**Table 1:** Estimated median age at first sex using survival (AFT) and traditional method by survey year and age groups

	N = 12,403^‡^	N = 4,069	N = 3,089	N = 2,369	N = 1,575	N = 7,537	N = 3,885	N = 3,060	N = 2,358	N = 1,573
	15–24 years	20–24 years	25–29 years	30–34 years	35–39 years	15–24 years	20–24 years	25–29 years	30–34 years	35–39 years
	Median from AFT^*^					Median from descriptive statistics (Traditional)^†^				
	n = 6,979	n = 2,665	n = 2,041	n = 1,469	n = 928	n = 4,599	n = 2,610	n = 2,037	n = 1,466	n = 925
*Females*										
Overall	16.92	16.88	16.70	16.67	16.57	16.00	17.00	17.00	17.00	16.00
*Years*										
1994–95	16.18	16.12	16.04	15.84	15.82	15.00	16.00	16.00	15.00	16.00
1996–97	16.64	16.57	15.55	15.99	15.81	16.00	16.00	15.00	16.00	16.00
1999–20	17.49	16.60	16.86	17.08	16.43	16.00	16.00	17.00	17.00	16.00
2003–04	16.36	16.80	16.75	16.97	16.57	16.00	17.00	17.00	17.00	17.00
2006–07	17.02	17.07	17.26	17.14	17.20	16.00	17.00	17.00	17.00	17.00
2010	17.09	17.06	17.03	17.13	17.08	16.00	17.00	17.00	17.00	17.00
2012–13	17.35	17.10	16.71	16.95	17.11	16.00	17.00	17.00	17.00	17.00
2015–16	17.61	17.69	17.44	17.02	17.19	17.00	18.00	18.00	17.00	17.00
*Males*										
	n = 5,424	n = 1,404	n = 1,048	n = 900	n = 651	n = 2,938	n = 1,275	n = 1,023	n = 892	n = 648
Overall	17.46	17.63	17.46	17.81	17.82	16.00	18.00	18.00	18.00	18.00
*Years*										
1994–95	16.98	17.14	17.17	17.49	17.35	16.00	17.00	17.00	18.00	18.00
1996–97	17.42	17.67	17.15	17.80	17.73	16.00	17.00	17.00	18.00	18.00
1999–20	18.18	18.77	17.27	17.37	17.90	15.00	17.00	17.00	17.00	18.00
2003–04	16.67	17.37	17.69	18.20	17.71	16.00	18.00	18.00	18.00	18.00
2006–07	16.81	17.20	17.15	17.29	17.71	16.00	17.00	17.00	17.00	18.00
2010	18.37	18.34	18.14	17.97	19.00	16.00	18.00	18.00	18.00	19.00
2012–13	18.30	18.45	18.09	17.98	18.43	16.00	18.00	18.00	18.00	18.00
2015–16	18.24	18.24	17.96	18.84	18.36	17.00	18.00	18.00	18.00	18.00

^**‡**^1087 (8.8%) reported first sex before 15 years; ^*^Take into account censoring (i.e., include those who never experienced the events); ^**†**^Estimated based only on those who experienced the events

**Fig. 3:**
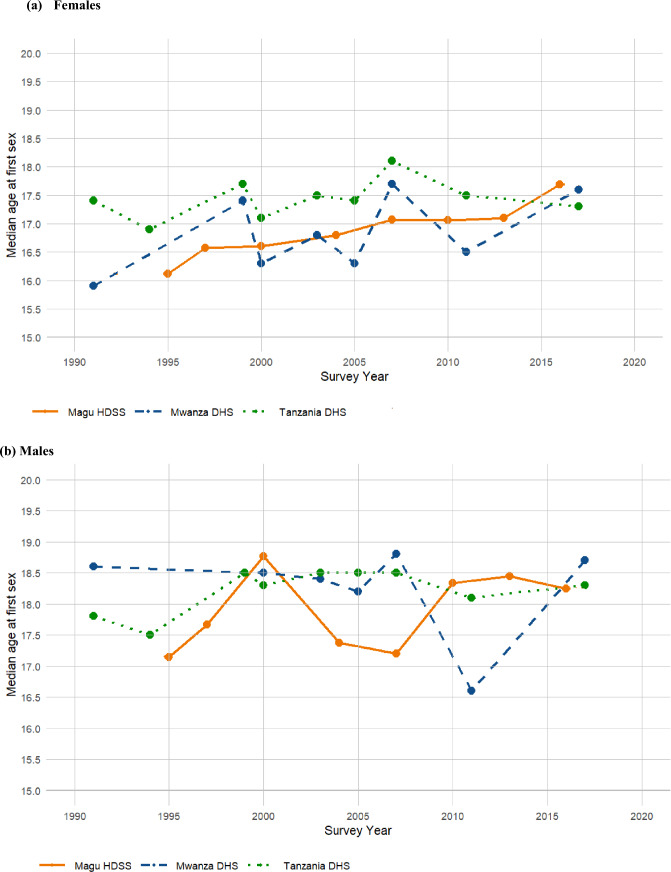
Trends in median age at first sex estimated with Magu HDSS, Mwanza and Tanzania DHS (20–24 years)

Figure 3a and b and Appendix: Table 2 present a comparison of the trends in median age at first sex estimated in this study (Magu HDSS) with those from Mwanza DHS and Tanzania DHS for females and males in the young adult age group (20–24 years). While erratic trends were observed for males in both surveys, for females, a systematic gradual increase in median age at first sex was noted for the Magu HDSS, rising from 16.12 years in 1995–17.69 years in 2016. In contrast, erratic trends were evident for females in Mwanza DHS and Tanzania DHS, with the median age at first sex fluctuating between 17.40 years in 1999 and 17.60 years in 2017 in Mwanza DHS and between 17.70 and 17.0 years in Tanzania DHS during the same period. In years where estimates are available across all surveys, such as 2000 and 2007, closely aligned and very slight differences in estimates between the Magu HDSS and Mwanza DHS. For instance, in 2000, the median age at first sex was 16.60 years in Magu HDSS compared to 16.30 years in Mwanza DHS, while in 2007, the median age at first sex was 17.07 years in Magu HDSS and 17.70 years in Mwanza DHS. These differences highlight the general consistency between the Magu HDSS and Mwanza DHS estimates, despite the erratic trends in other surveys (Fig. 3a and Appendix: Table 2).

**Table 2:** Comparison of median age at first sex estimated by Magu HDSS (Kisesa) and DHS for females and males by survey years and age group

	20–24 years			25–29 years			30–34 years			35–39 years		
Survey year	Magu HDSS	Mwanza DHS	Tanzania DHS	Magu HDSS	Mwanza DHS	Tanzania DHS	Magu HDSS	Mwanza DHS	Tanzania DHS	Magu HDSS	Mwanza DHS	Tanzania DHS
*Females*												
1991		15.90	17.40		16.10	16.90		15.90	16.90		15.30	16.40
1994			16.90			16.80			16.80			16.30
1995	16.12			16.04			15.84			15.82		
1997	16.57			15.55			15.99			15.81		
1999		17.40	17.70		16.90	17.60		17.00	17.70		17.60	17.60
2000	16.60	16.30	17.10	16.86	16.70	17.30	17.08	16.40	17.30	16.43	16.50	17.00
2003		16.80	17.50		17.30	17.50		17.30	17.50		16.40	17.00
2004	16.80			16.75			16.97			16.57		
2005		16.30	17.40		16.60	17.40		16.20	17.40		16.60	17.30
2007	17.07	17.70	18.10	17.26	16.80	17.90	17.14	17.70	17.90	17.20	17.40	17.90
2010	17.06			17.03			17.13			17.08		
2011		16.50	17.50		16.90	17.40		16.40	17.40		16.00	17.20
2013	17.10			16.71			16.95			17.11		
2016	17.69			17.44			17.02			17.19		
2017		17.60	17.30		17.00	17.10		16.00	17.10		16.30	16.80
*Males*												
Survey year	Magu HDSS	Mwanza DHS	Tanzania DHS	Magu HDSS	Mwanza DHS	Tanzania DHS	Magu HDSS	Mwanza DHS	Tanzania DHS	Magu HDSS	Mwanza DHS	Tanzania DHS
1991		18.60	17.80		18.00	17.60		18.80	18.10		19.10	18.20
1994			17.50			17.60			17.60			17.70
1995	17.14			17.17			17.49			17.35		
1997	17.67			17.15			17.80			17.73		
1999		18.50	18.50		18.20	18.60		18.10	18.70		18.40	18.70
2000	18.77	18.50	18.30	17.27	17.50	18.20	17.37	18.50	18.40	17.90	16.20	18.70
2003		18.40	18.50		18.40	18.50		18.00	18.50		18.20	18.50
2004	17.37			17.69			18.20			17.71		
2005		18.20	18.50		17.30	18.20		18.30	18.80		16.90	18.50
2007	17.20	18.80	18.50	17.15	18.20	18.60	17.29	18.40	18.80	17.71	18.50	18.90
2010	18.34			18.14			17.97			19.00		
2011		16.60	18.10		19.70	18.30		17.30	17.90		16.20	18.10
2013	18.45			18.09			17.98			18.43		
2016	18.24			17.96			18.84			18.36		
2017		18.70	18.30		17.70	18.20		18.90	18.70		19.00	18.70

### Age at First Sex by Education and HIV Status

**Fig. 4:**
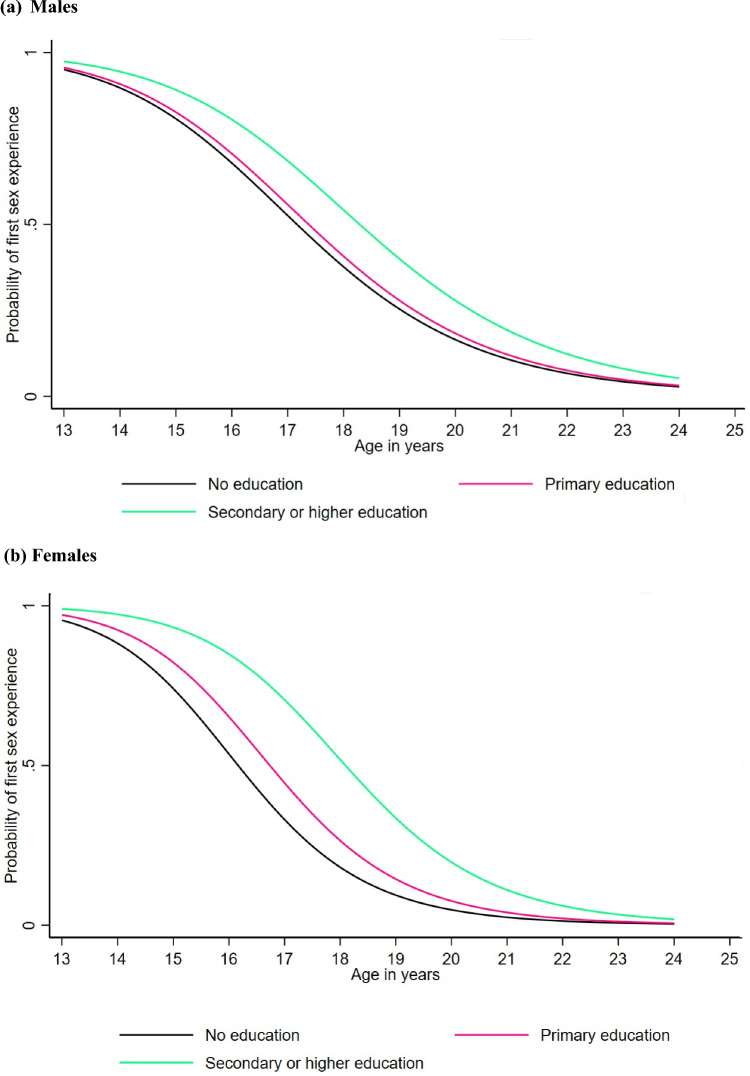
Estimated probability of first sex experience for the log-logistic AFT model for males and females by education level

**Fig. 5:**
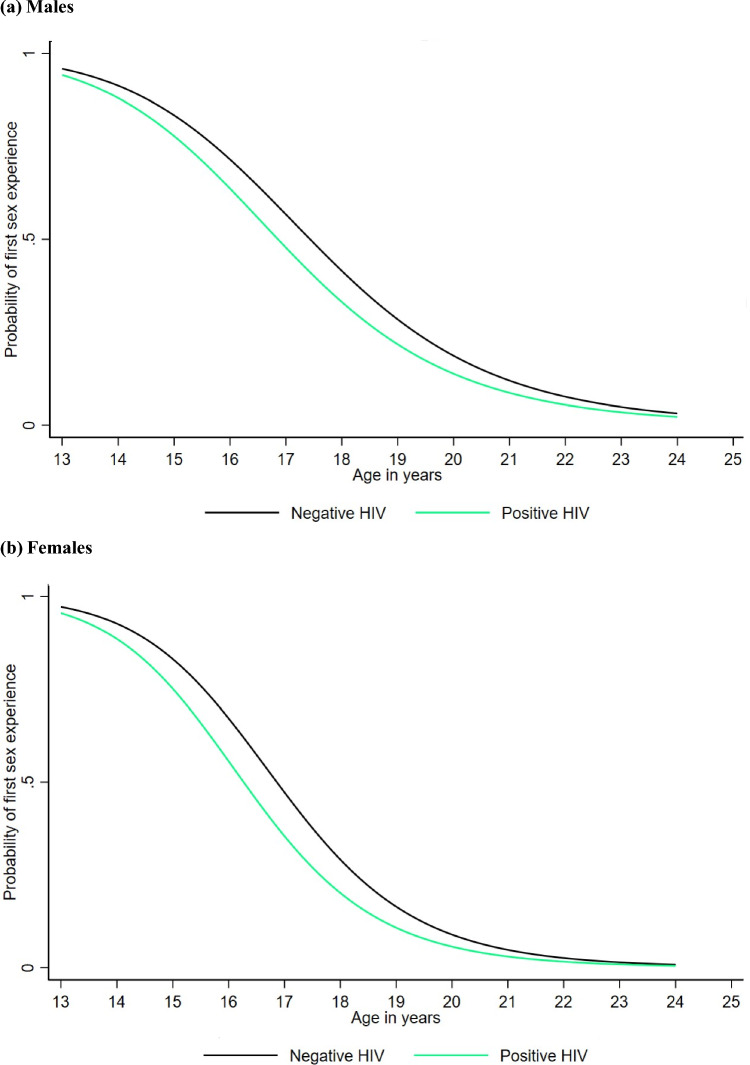
Estimated probability of first sex experience for the log-logistic AFT model for males and females by HIV status

The median age at first sex was significantly lower for males and females with no education compared to those with secondary or higher education (Fig. 4a and b). For males, the median age was 17.1 years for those with no education compared to 18.3 years for those with secondary or higher education, while for females, it was 16.2 years and 18.2 years, respectively. Overall, females and males with no education experience first sex at a younger age compared to those with secondary or higher education, although the difference starts to become minimal or insignificant from 22 years onwards. A very small and insignificant difference was also observed between males with no education (17.1 years) and those with primary education (17.3 years) (Fig. 4a). Additionally, males and females with a positive HIV status experienced first sex slightly earlier (16.9 years and 16.3 years, respectively) compared to those with a negative HIV status (17.5 years and 16.9 years, respectively) (Fig. 5a and b), but this difference minimized from 22 years onwards.

### Trend in Age at First Marriage

**Fig. 6:**
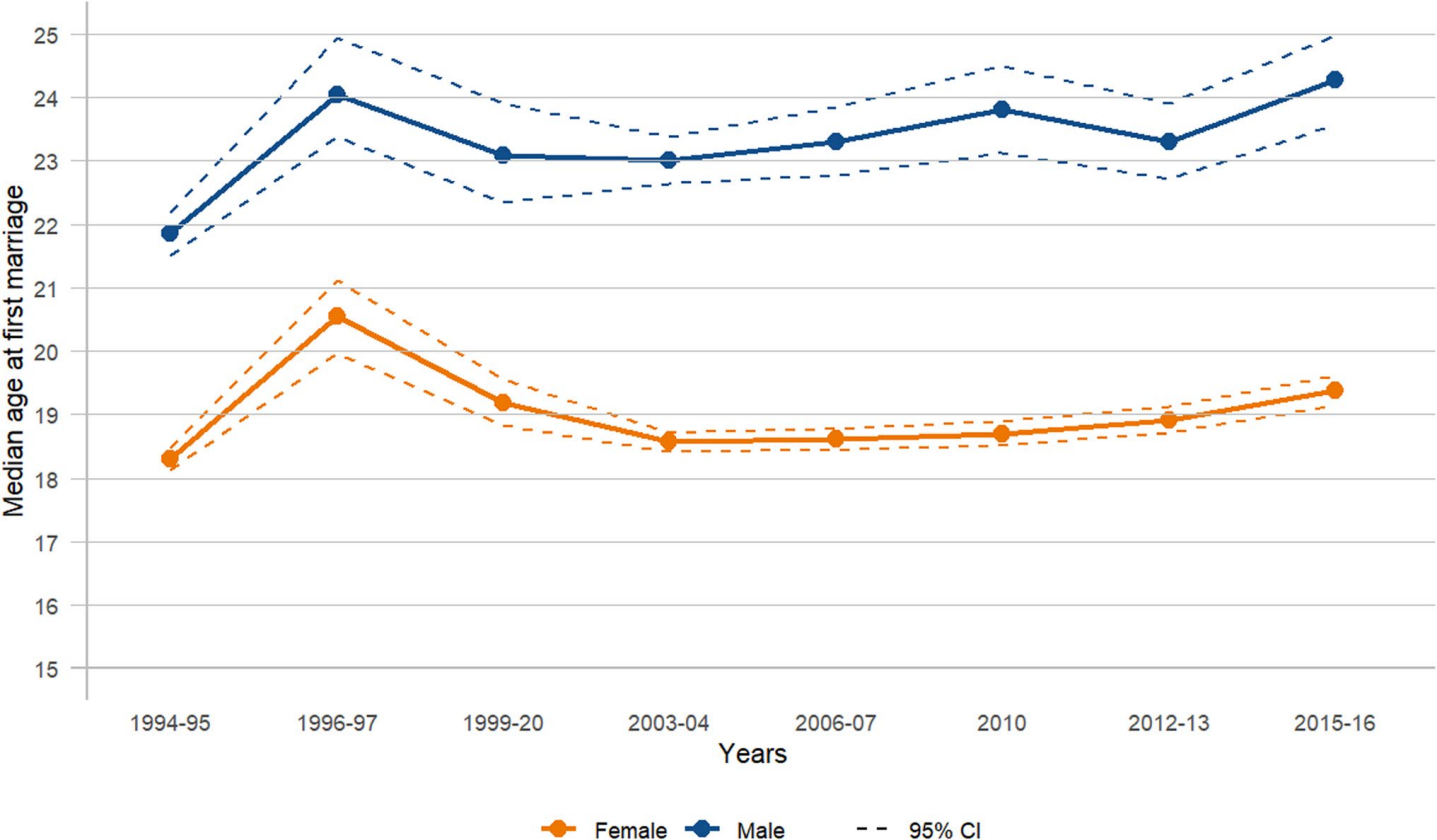
Trends in median age at first marriage by survey year, females and males (15–24 years)

**Fig. 7:**
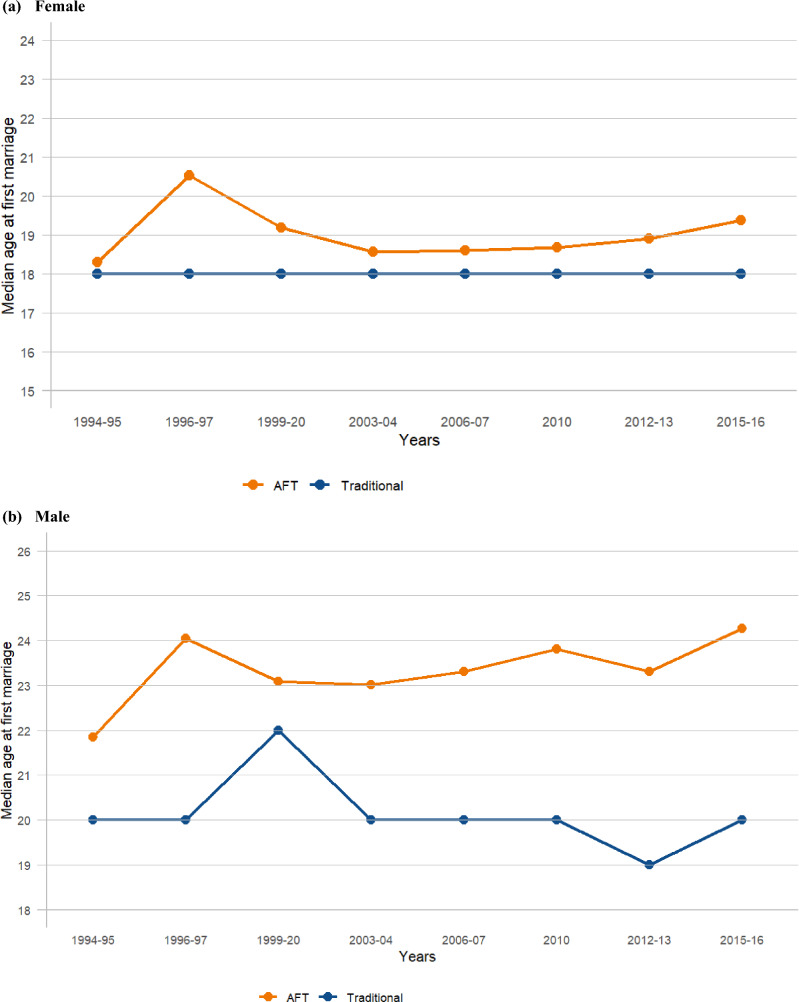
Trends in median age at first marriage estimated using AFT and traditional methods (15–24 years)

From 1994–1995 to 2015–2016, the median age at first marriage increased by approximately one year for females (from 18.30 years in 1994–1995 to 19.38 years in 2015–2016) and two years for males (from 21.85 years in 1994–1995 to 24.27 years in 2015–2016), although this progression was not consistent annually (Fig. 6 and Appendix: Table 3). There was a slight systematic rise observed from 2003–04 to 2015–16 for females (from 18.57 years in 2003–2004 to 19.38 years in 2015–2016) and from 2003–04 to 2010 for males (from 23.01 years in 2003–2004 to 23.81 years in 2010). Overall, males consistently had a higher median age at first marriage compared to females across all survey years. The year 1996–1997 displayed slightly elevated values for both sexes, with a median age at first marriage of 20.54 years for females and 24.04 years for males, compared to the surrounding years. When comparing the trends in estimated median age at first marriage using the AFT model with those derived from the traditional method (standard descriptive statistics), similar trends were observed as in age at first sex. The overall median age at first marriage was higher with the AFT model for both females and males. Moreover, the AFT model was better at capturing subtle changes compared to the standard descriptive statistics, which exhibited no changes (a constant or flat trend) in certain years for both sexes (females: 18.00–18.00 from 1994–1995 to 2015–2016; males: 20.00–20.00 from 2003–2004 to 2010) (Fig. 7a and b; Appendix: Table 3).

**Table 3:** Comparison of median age at first marriage using survival (AFT) and traditional method by survey year and age groups

	N = 11,116^‡^	N = 4,051	N = 3,054	N = 2,389	N = 1,581	N = 3,484	N = 2,622	N = 2,678	N = 2,258	N = 1,529
	15–24 years	20–24 years	25–29 years	30–34 years	35–39 years	15–24 years	20–24 years	25–29 years	30–34 years	35–39 years
	Median from AFT^*^					Median from descriptive statistics (Traditional)^†^				
	n = 6,492	n = 2,664	n = 2,018	n = 1,459	n = 898	n = 3,048	n = 2,234	n = 1,913	n = 1,414	n = 875
*Females*										
Overall	18.79	18.90	18.86	18.63	18.22	18.00	18.00	18.00	18.00	18.00
*Years*										
1994–95	18.30	18.51	18.46	17.82	16.98	18.00	18.00	18.00	18.00	17.00
1996–97	20.54	20.55	19.88	19.26	17.95	18.00	20.00	18.00	19.00	18.00
1999–20	19.19	19.08	19.06	18.82	18.22	18.00	18.00	18.00	18.00	18.00
2003–04	18.57	18.99	18.98	18.85	18.66	18.00	19.00	18.50	19.00	18.50
2006–07	18.61	18.72	19.04	19.18	18.68	18.00	18.00	19.00	19.00	18.50
2010	18.69	18.70	18.80	18.67	18.74	18.00	18.00	19.00	18.00	19.00
2012–13	18.91	19.04	18.64	19.17	19.02	18.00	18.00	18.00	19.00	18.00
2015–16	19.38	19.31	19.29	18.62	18.57	18.00	18.00	18.00	18.00	18.00
*Males*										
	n = 4,624	n = 1,387	n = 1,036	n = 930	n = 683	n = 436	n = 388	n = 765	n = 844	n = 654
Overall	23.33	23.48	23.88	24.69	24.37	20.00	20.00	22.00	24.00	24.00
*Years*										
1994–95	21.85	21.93	22.84	24.21	24.12	20.00	20.00	22.00	24.00	24.00
1996–97	24.04	23.24	28.75	27.65	25.50	20.00	22.00	23.00	23.00	25.00
1999–20	23.09	23.07	26.49	26.11	23.96	22.00	22.00	24.00	24.00	24.00
2003–04	23.01	23.14	23.90	25.58	24.59	20.00	21.00	22.00	25.00	25.00
2006–07	23.31	23.58	23.21	24.17	24.21	20.00	20.00	23.00	24.00	24.50
2010	23.81	24.00	24.40	23.76	25.03	20.00	20.00	23.00	24.00	25.00
2012–13	23.31	23.34	24.43	25.41	24.39	19.00	20.00	23.00	25.00	24.00
2015–16	24.27	24.56	24.19	24.43	23.97	20.00	20.00	22.00	24.00	24.00

^**‡**^6112 (55.0%) reported first marriage before 18 years; ^*^Take into account censoring (i.e., include those who never experienced the events); ^**†**^Estimated based only on those who experienced the events

**Fig. 8:**
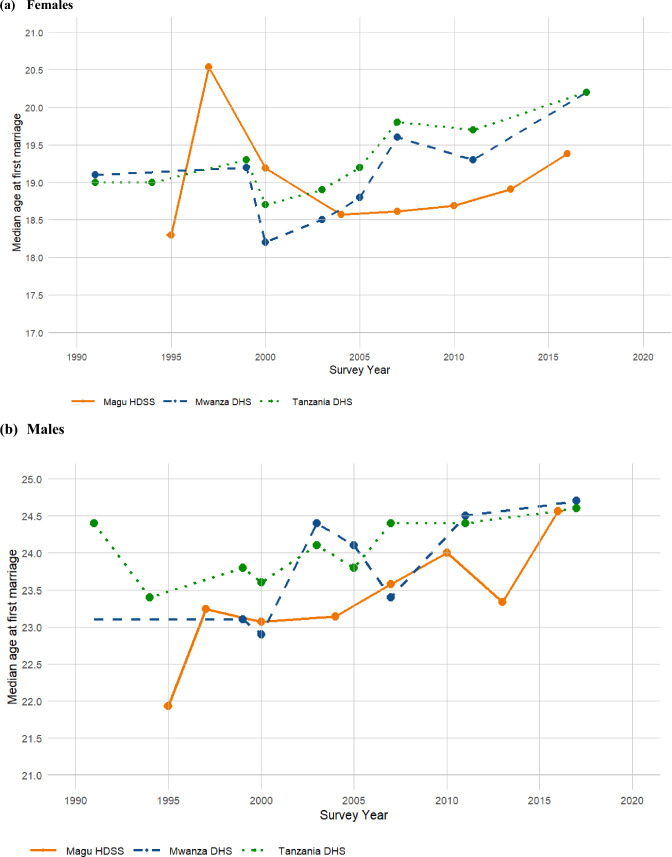
Trends in median age at first marriage estimated with Magu HDSS, Mwanza and Tanzania DHS (20–24 years)

Figure 8a and b and Appendix: Table 4 show a comparison between the trends in median age at first marriage as estimated in our study (Magu HDSS) and those from Mwanza DHS and Tanzania DHS. This comparison focuses on females and males in the young adult age group (20–24 years). While Mwanza and Tanzania DHS surveys displayed erratic trends across most years, Magu HDSS data showed a consistent gradual increase in median age at first marriage for females, from 18.72 years in 2007 to 19.31 years in 2016, and for males, from 23.07 years in 2000 to 24.00 years in 2010.

**Table 4:** Comparison of median age at first marriage estimated by Magu HDSS (Kisesa) and DHS for females and males by survey years and age group

	20–24 years			25–29 years			30–34 years			35–39 years		
Survey year	Magu HDSS	Mwanza DHS	Tanzania DHS	Magu HDSS	Mwanza DHS	Tanzania DHS	Magu HDSS	Mwanza DHS	Tanzania DHS	Magu HDSS	Mwanza DHS	Tanzania DHS
*Females*												
1991		19.10	19.00		18.80	18.70		19.30	18.50		16.70	17.60
1994			19.00			18.80			18.50			18.00
1995	18.51			18.46			17.82			16.98		
1997	20.55			19.88			19.26			17.95		
1999		19.20	19.30		19.20	19.10		19.20	18.70		18.60	18.80
2000	19.08	18.20	18.70	19.06	18.50	19.00	18.82	18.40	18.70	18.22	17.70	18.40
2003		18.50	18.90		18.30	19.00		19.70	19.10		17.80	18.60
2004	18.99			18.98			18.85			18.66		
2005		18.80	19.20		19.10	18.90		18.80	19.10		19.00	18.60
2007	18.72	19.60	19.80	19.04	19.50	19.40	19.18	18.00	19.20	18.68	19.50	19.10
2010	18.70			18.8			18.67			18.74		
2011		19.30	19.70		19.70	19.60		18.40	19.10		19.80	19.20
2013	19.04			18.64			19.17			19.02		
2016	19.31			19.29			18.62			18.57		
2017					20.20	20.20		20.40	20.40		19.10	19.40
*Males*												
1991						24.40		24.50	24.40		25.50	25.30
1994						23.40			23.80			24.30
1995	21.93			22.84			24.21			24.12		
1997	23.24			22.75			27.65			25.50		
1999					23.10	23.80		24.20	24.20		24.90	24.10
2000	23.07			23.49	22.90	23.60	26.11	24.40	24.60	23.96	23.90	24.40
2003					24.40	24.10		22.10	23.80		23.90	24.80
2004	23.14			23.90			25.58			24.59		
2005					24.10	23.80		22.80	23.90		22.80	24.60
2007	23.58			23.21	23.40	24.40	24.17	26.70	24.70	24.21	25.30	24.30
2010	24.00			24.40			23.76			25.03		
2011						24.40		24.50	24.50		23.90	23.80
2013	23.34			24.43			25.41			24.39		
2016	24.56			24.19			24.43			23.97		
2017						24.60		24.70	25.20		26.00	24.80

### Age at First Marriage by Education and HIV Status

**Fig. 9:**
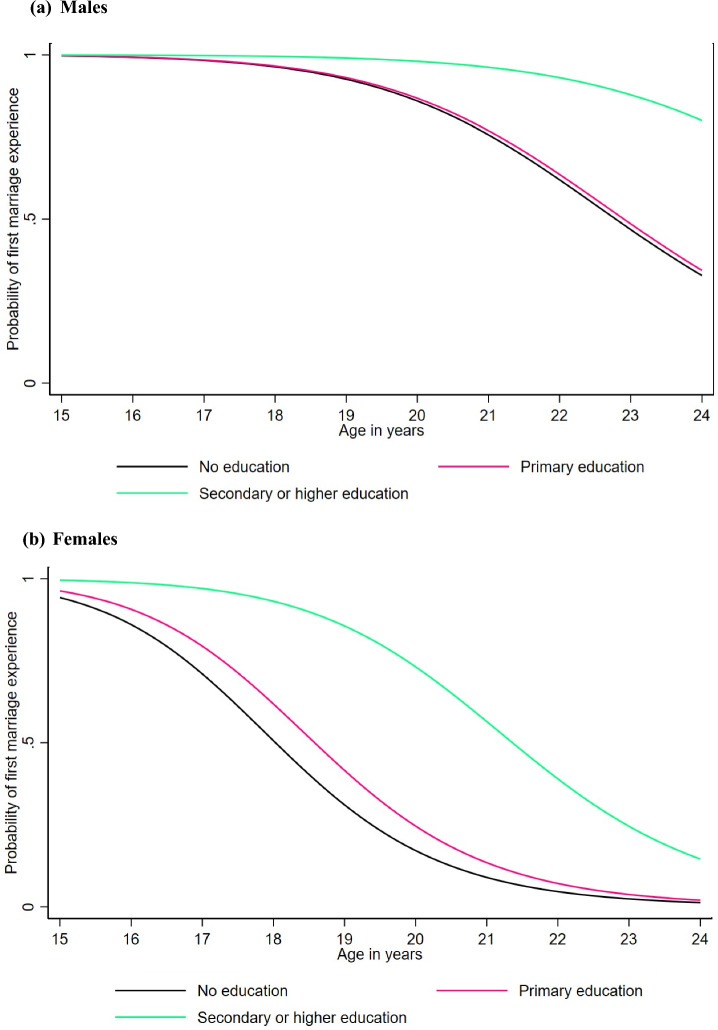
Estimated probability of first marriage experience for the log-logistic AFT model for males and females by education level

**Fig. 10:**
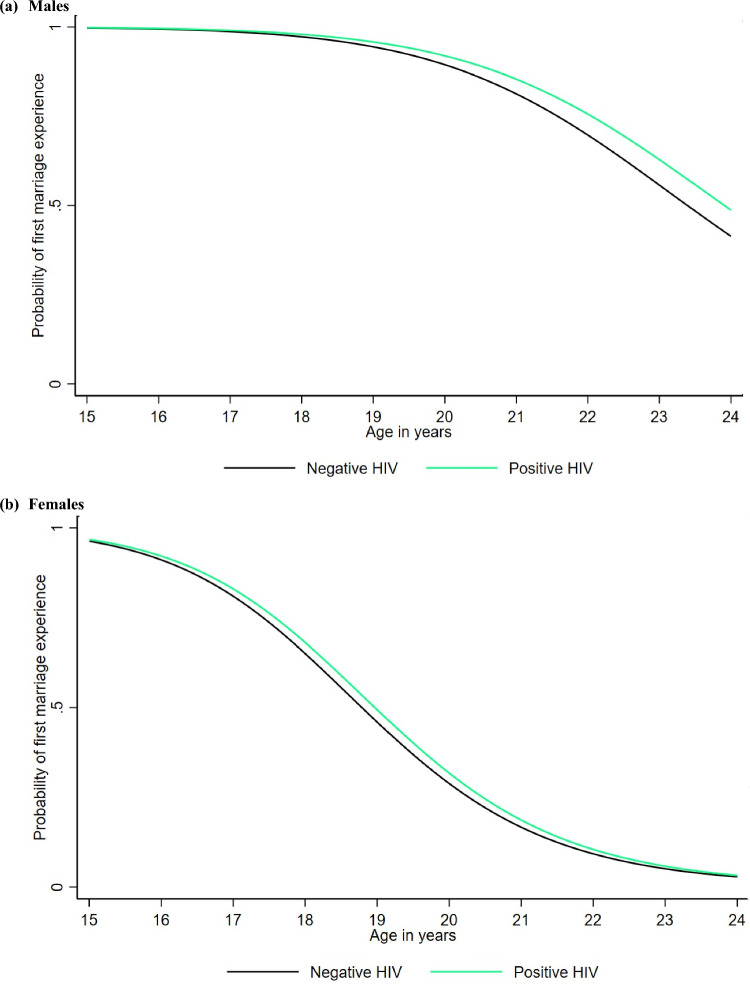
Estimated probability of first marriage experience for the log-logistic AFT model for males and females by HIV status

Figure 9 shows the estimated probability of age at first marriage by education level. For males, no difference was observed in age at first marriage between those with no education (median: 22.8 years) and primary education (median: 22.9 years), while those with secondary education or higher experienced later age at first marriage, with the median age not reached by the end of the observed age period (Fig. 9a). For females, the median age at first marriage was significantly lower for those with no education (18.0 years) compared to those with secondary or higher education (21.3 years). The difference between females with no education and those with primary education was smaller (18.0 years vs. 18.7 years) and very minimal from age 22 onwards (Fig. 9b). Additionally, males with a positive HIV status experienced first marriage slightly later (median: 23.9 years) compared to those with a negative HIV status (median: 23.4 years), while for females the difference was minimal (negative: 18.8 years vs. positive: 19.0 years) (Fig. 10a and b).

## Discussion

The findings of this study provide valuable insights into the trends of the median age at first sex and the age at first marriage using the AFT model among the young population aged 15–24 in Magu HDSS, Tanzania. Overall, the median age at first sex increased by one year for both sexes, while for age at first marriage, it increased by one year for females and two years for males. Males exhibited higher median ages at first sex and at first marriage compared with females. The comparison between the medians estimated using the survival method (AFT model) and the traditional method (median from standard descriptive statistics) showed higher median ages at first sex and at first marriage from the AFT model compared with the traditional method.

The AFT model captured small changes and showed gradual increases from 2003–2004 to 2015–2016 for females and from 2003–2004 to 2010 for males, while the traditional method showed no changes (a constant or flat trend) in some years for both females and males. Specifically, the years showing no changes were: for median age at first sex, 1996–1997 to 2012–2013 for females, and 2003–2004 to 2012–2013 for males; and for median age at first marriage, 1994–1995 to 2015–2016 for females, and 2003–2004 to 2010 for males. This ability to capture subtle variations enhances the AFT model’s utility in discerning dynamic trends in age at first sex and age at first marriage data while fully utilizing all data sets (i.e., accounting for censoring). By employing the AFT model, we have enhanced the ability to use all self-reported data for the young population without omitting those who never experienced the events or the adolescent age group (15–19 years) during the estimation. This approach offers valuable insights that can inform public health policies and interventions. In real life, a substantial number of young people experience these events before age 20, so omitting them during analysis is not a good approach. For example, in this study (Magu HDSS), among the young population aged 15–24 years, 8.8% reported first sex before age 15 and 55% reported first marriage before age 18 (Appendix: Table 1 and Table 3). We also observed an increase in the median age at first sex in the early years (1994–2000) for both females and males before it dropped again for both sexes. This period coincided with the height of the HIV epidemic in this area, and the increase in median age at first sex around this time might be associated with efforts to abstain from sexual activity and potential HIV fears (Urassa et al., 2024).

When comparing median age at first sex and age at first marriage trends with those from the Mwanza DHS and Tanzania DHS (TDHS) for young adults (20–24 years) of both sexes, we observed erratic trends across the years for Mwanza and TDHS, while systematic gradual changes were observed in certain years in Magu HDSS. Notable closeness in estimates was observed during specific periods, such as 2000 and 2007 for the median age at first sex among females and 2000 for males, highlighting that the Magu HDSS data is a good representation of the population in Tanzania (Urassa et al., 2024). The observed discrepancies may stem from differences in sample sizes, methodologies, or population characteristics between our study and the TDHS.

When stratified by education level, our results showed that individuals with no education had significantly lower median ages at first sex and at first marriage compared to those with secondary or higher education. Specifically, for males, the median age at first sex was 17.1 years for those with no education compared to 18.3 years for those with secondary or higher education, while for females, it was 16.2 years versus 18.2 years, respectively. Similarly, for the median age at first marriage, the median age for females was 18.0 years for those with no education compared to 21.3 years for those with secondary or higher education. This pattern was consistent for both males and females, emphasizing the role of education in delaying the onset of sexual activity and marriage. Additionally, those with a positive HIV status experienced first sex and marriage slightly earlier than those with a negative status, although the difference was very minimal for both genders. For males, the median age at first sex was 17.5 years for those with a negative status compared to 16.9 years for those with a positive status, while for females, the median age was 18.8 years for those with a negative status and 19.0 years for those with a positive status. The increase in educational attainment over the study period has likely contributed to the observed delays in the median age at first sex and median age at first marriage. The substantial rise in education levels reflects broader societal changes and the effectiveness of educational policies implemented during this period. This compositional change in education is a crucial factor in understanding the dynamics of sexual and marital timing among young people. As more individuals attain higher education, the overall trends shift toward later ages for these milestones, underscoring the significant role of education in shaping sexual and reproductive behaviors. Our findings align with global patterns indicating that higher educational attainment is associated with later age at first sex and age at first marriage. Studies from various regions, including sub-Saharan Africa, have documented similar trends, reinforcing the protective role of education in delaying these milestones (Hargreaves et al., 2012; Ikamari, 2005; Lindstrom et al., 2022; Marphatia et al., 2020; Reda & Lindstrom, 2014).

The analysis of the median age at first sex and median age at first marriage reveals significant periods of premarital sexual activity among the young population in Magu, Tanzania, ranging from 1.6 to 3.9 years for females and 4.9 to 6.6 years for males (Appendix: Table 5). These findings underscore the importance of addressing contraceptive use and associated risks during this premarital period. Prolonged premarital sexual activity without adequate contraceptive use increases the risk of unintended pregnancies and sexually transmitted infections (STIs), which can have long-term social and economic consequences (Bertrand et al., 2023; Das & Rout, 2023). Therefore, public health policies should emphasize comprehensive sexual education and access to contraceptive methods for young, unmarried individuals (Krugu & van der Kwaak, 2024). Addressing the social stigma around premarital sex and contraceptive use is also crucial to ensure young people can make informed decisions about their sexual health (Krugu & van der Kwaak, 2024).

**Table 5:** Years of premarital sex for females and males

Years	Median AFS Female	Median AFS Male	Median AFM Female	Median AFM Male	Years of premarital sex Female	Years of premarital sex Male
1995	16.18	16.98	18.3	21.85	2.10	4.90
1997	16.64	17.42	20.54	24.04	3.90	6.60
2000	17.49	18.18	19.19	23.09	1.70	4.90
2004	16.36	16.67	18.57	23.01	2.20	6.30
2007	17.02	16.81	18.61	23.31	1.60	6.50
2010	17.09	18.37	18.69	23.81	1.60	5.40
2013	17.35	18.3	18.91	23.31	1.60	5.00
2016	17.61	18.24	19.38	24.27	1.80	6.00

AFS = Age at first sex; AFM = Age at first marriage

We also observed slightly earlier experiences of first sex or marriage when utilizing data corrected for inconsistencies compared to uncleaned data (Appendix: Fig. 1). This suggests that many of the inconsistencies may be attributable to random misreporting rather than systematic biases. This slight difference indicates that the observed trends can be interpreted with some confidence for uncorrected data as well, despite some levels of misreporting. Furthermore, this provides some reassurance when interpreting trends in serial cross-sectional data, such as from Demographic and Health Surveys (DHS), where such inconsistencies cannot be identified. However, consistency of reporting does not always signify accuracy; an individual may consistently report an incorrect age at first sex, a bias which cannot be detected. This small difference, which overall had an insignificant impact, has also been observed in another study (Cremin et al., 2009; Upchurch et al., 2002). However, it is always worth practicing good methodology to correct and clean data for estimating trends and other analyses, as the significant impact may not be evident when dealing with a large sample size, but the story can be different when dealing with a small sample size.

Our findings have some implications for public health interventions and policies aimed at promoting sexual and reproductive health among young population in Tanzania. The observed increase in the median age at first sex and median age at first marriage underscores the need for comprehensive sexuality education and access to reproductive health services to ensure informed decision-making and safe practices among adolescents and young adults. Moreover, addressing disparities in sexual initiation by education level calls for targeted interventions tailored to the needs of vulnerable populations, particularly those with limited access to education.

One of the limitations of this study is the potential presence of anomalies or external influences in certain years, which could introduce bias into the observed trends in median age at first sex and median age at first marriage. These anomalies, particularly notable in 1999–2000 for age at first sex and 1996–1997 for age at first marriage, may reflect the influence of unaccounted factors, such as programs promoting sexual abstinence and other HIV interventions aimed at reducing HIV prevalence (Urassa et al., 2024). Additionally, the surveys did not distinguish between formal marriage and cohabitation. This conflation may introduce bias in estimating median age at first marriage across subgroups, as behaviors may differ between formal marriage and cohabiting individuals. Despite this limitation, the overall trends observed are likely robust, especially when using corrected and cleaned data across multiple survey rounds. Also, despite efforts to correct inconsistencies in the data, it is challenging to ascertain the accuracy of values reported by single respondents across surveys, unlike for those who appeared in multiple rounds, potentially impacting the precision of the estimates. However, despite this limitation, the study spans a considerable time period from 1994–95 to 2015–16, enabling the examination of trends over time. This longitudinal analysis provides valuable insights into changes in age at first sex and age at first marriage over the years among the young population (aged 15–24) using all available data.

In conclusion, access to education remains a significant factor in delaying these events, especially the age at first marriage. There is no significant difference affecting the trend in the median of these events or the inference between the corrected and uncorrected data. However, it is always worthwhile to correct the data to increase confidence in the final estimates and inference of the results. Additionally, using AFT model as a parametric method under survival analysis to estimate the median is useful, as it allows us to utilize all data in the estimation process and capture even small changes rather than relying only on traditional descriptive statistics.

## Electronic supplementary material

Below is the link to the electronic supplementary material.

Supplementary file1 (XLS 4761 KB)

## Figures and Tables

**Fig. 11:**
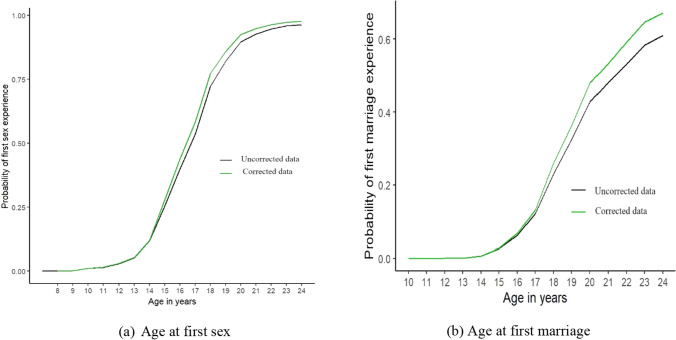
Kaplan–Meier failure curve for age at first sex and marriage of the young population (15–24 years) before and after the correction of inconsistency

## Data Availability

All the data supporting the findings presented in this manuscript are included as supplementary information.

## Appendix

See Figure 11.

## Code Availability

Code is available upon request.

## References

[CR1] Adair, T. (2008). HIV status and age at first marriage among women in Cameroon. *Journal of Biosocial Science,**40*(5), 743–760. 17988430 10.1017/S0021932007002556

[CR2] Ajaegbu, O. O. (2015). Premarital sex, HIV, and use of condom among youths in Nigeria. *International Journal of Psychological and Behavioral Sciences,**9*(12), 4133–4137.

[CR3] Becker, M. L., Bhattacharjee, P., Blanchard, J. F., Cheuk, E., Isac, S., Musyoki, H. K., Gichangi, P., Aral, S., Pickles, M., & Sandstrom, P. (2018). Vulnerabilities at first sex and their association with lifetime gender-based violence and HIV prevalence among adolescent girls and young women engaged in sex work, transactional sex, and casual sex in Kenya. *JAIDS Journal of Acquired Immune Deficiency Syndromes,**79*(3), 296–304. 30113403 10.1097/QAI.0000000000001826 PMC6203425

[CR4] Bertrand, J. T., Ross, J. A., & Sauter, S. R. (2023). Trends in contraceptive method mix among adolescents and youth aged 15–24 in low-and middle-income countries. *Frontiers in Global Women’s Health,**3*, 1061648. 36713979 10.3389/fgwh.2022.1061648 PMC9875564

[CR43] Cohen, B., & Bessinger, R. (2003). *Sexual behavior, HIV, and fertility trends: A comparative analysis of six countries. MEASURE Evaluation*. https://www.measureevaluation.org/resources/publications/sr-03-21b.html

[CR5] Cremin, I., Mushati, P., Hallett, T., Mupambireyi, Z., Nyamukapa, C., Garnett, G., & Gregson, S. (2009). Measuring trends in age at first sex and age at marriage in Manicaland. *Zimbabwe. Sexually Transmitted Infections,**85*(Suppl 1), i34–i40. 19307339 10.1136/sti.2008.033431 PMC2654143

[CR6] Das, U., & Rout, S. (2023). Are delay ages at marriage increasing? Pre-marital sexual relation among youth people in the place of residence in India. *BMC Women’s Health,**23*(1), 16. 36631806 10.1186/s12905-022-02149-3 PMC9835306

[CR7] Eaton, J. W. (2022). A model for reconstructing trends and distribution in age at first sex from multiple household surveys with reporting biases. *Epidemics,**40*, Article 100593. 35785637 10.1016/j.epidem.2022.100593 PMC9469639

[CR8] Fagbamigbe, A. (2021). How soon does modern contraceptive use starts after sexual debut in Africa? Survival analysis of timing and associated factors among never-in-union women. *Scientific African,**11*, Article e00719.

[CR10] Gupta, N., & Mahy, M. (2003). Sexual initiation among adolescent girls and boys: Trends and differentials in sub-Saharan Africa. *Archives of Sexual Behavior,**32*, 41–53. 12597271 10.1023/a:1021841312539

[CR11] Hargreaves, J. R., Slaymaker, E., Fearon, E., & Howe, L. D. (2012). Changes over time in sexual behaviour among young people with different levels of educational attainment in Tanzania. *Journal of the International AIDS Society,**15*, 17363. 10.7448/IAS.15.3.17363 10.7448/IAS.15.3.17363 PMC3499906 22713351

[CR12] ICF. (2012). *The DHS Program STATcompiler*. Funded by USAID. Retrieved 06/02/2024 from http://www.statcompiler.com

[CR13] Ikamari, L. D. (2005). The effect of education on the timing of marriage in Kenya. *Demographic Research,**12*. https://www.demographic-research.org/volumes/vol12/1/12-1.pdf

[CR14] Krugu, J. K., & van der Kwaak, A. (2024). *Adolescent sexual and reproductive health in low- and middle-income countries: A synthesis of research findings for improved program development and implementation*. KIT Royal Tropical Institute. https://www.kit.nl/wp-content/uploads/2019/05/Adolescent-Research-in-Brief_Sida.docx

[CR15] Lindstrom, D. P., Sahlu, I., Belachew, T., & Gerbaba, M. (2022). Life expectations in early adolescence and the timing of first sex and marriage: Evidence from a longitudinal survey in Ethiopia. *Reproductive Health,**19*(Suppl 1), 196. 35698147 10.1186/s12978-021-01239-z PMC9195193

[CR16] Magadi, M. A., & Agwanda, A. O. (2010). Investigating the association between HIV/AIDS and recent fertility patterns in Kenya. *Social Science & Medicine,**71*(2), 335–344. 10.1016/j.socscimed.2010.03.040 20494502 10.1016/j.socscimed.2010.03.040

[CR17] Magnusson, B. M., Masho, S. W., & Lapane, K. L. (2012). Early age at first intercourse and subsequent gaps in contraceptive use. *Journal of Women’s Health,**21*(1), 73–79. 21992618 10.1089/jwh.2011.2893 PMC3283439

[CR18] Marphatia, A. A., Saville, N. M., Amable, G. S., Manandhar, D. S., Cortina-Borja, M., Wells, J. C., & Reid, A. M. (2020). How much education is needed to delay women’s age at marriage and first pregnancy? *Frontiers in Public Health,**7*, Article 495704. 10.3389/fpubh.2019.00396 PMC6964653 31993411

[CR19] Marston, M., Zaba, B., & Eaton, J. W. (2017). The relationship between HIV and fertility in the era of antiretroviral therapy in sub-Saharan Africa: Evidence from 49 demographic and health surveys. *Tropical Medicine & International Health,**22*(12), 1542–1550. 28986949 10.1111/tmi.12983 PMC5716842

[CR20] Materu, J., Konje, E. T., Urassa, M., Marston, M., Boerma, T., & Todd, J. (2023). Comparison of survival analysis approaches to modelling age at first sex among youth in Kisesa Tanzania. *PLoS ONE,**18*(9), Article e0289942. 37676876 10.1371/journal.pone.0289942 PMC10484422

[CR21] Materu, J., Todd, J., Slaymaker, E., Urassa, M., Marston, M., Boerma, T., & Konje, E. T. (2025). Consistency in self-reported age at first sex and marriage among adolescents and young adults in Northwestern Tanzania: Insights from repeated responses. *Frontiers in Reproductive Health,**7*, 1488604. 40574903 10.3389/frph.2025.1488604 PMC12198193

[CR22] Mensch, B. S., Grant, M. J., & Blanc, A. K. (2006). The changing context of sexual initiation in sub-Saharan Africa. *Population and Development Review, 32*, 699–727. 10.1111/j.1728-4457.2006.00147.x

[CR23] Mensch, B. S., Hewett, P. C., & Erulkar, A. S. (2003). The reporting of sensitive behavior by adolescents: A methodological experiment in Kenya. *Demography,**40*(2), 247–268. 12846131 10.1353/dem.2003.0017

[CR24] Mensch, B. S., Soler-Hampejsek, E., Kelly, C. A., Hewett, P. C., & Grant, M. J. (2014). Challenges in measuring the sequencing of life events among adolescents in Malawi: A cautionary note. *Demography,**51*(1), 277–285. 24399140 10.1007/s13524-013-0269-2 PMC4175924

[CR25] Ministry of Health, Community Development, Gender, Elderly and Children (MoHCDGEC) [Tanzania Mainland]; Ministry of Health (MoH) [Zanzibar]; National Bureau of Statistics (NBS); Office of the Chief Government Statistician (OCGS); & ICF. (2016). *Tanzania Demographic and Health Survey and Malaria Indicator Survey (TDHS-MIS) 2015–16*. Dar es Salaam, Tanzania, and Rockville, Maryland, USA: MoHCDGEC, MoH, NBS, OCGS, and ICF. https://dhsprogram.com/pubs/pdf/FR321/FR321.pdf

[CR26] Ministry of Health (MoH) [Tanzania Mainland], Ministry of Health (MoH) [Zanzibar], National Bureau of Statistics (NBS), Office of the Chief Government Statistician (OCGS), & ICF. (2022). *Tanzania Demographic and Health Survey and Malaria Indicator Survey 2022 Final Report*. https://dhsprogram.com/pubs/pdf/PR144/PPR144.pdf

[CR27] Nahar, M. Z., Zahangir, M. S., & Shafiqul Islam, S. (2013). Age at first marriage and its relation to fertility in Bangladesh. *Chinese Journal of Population Resources and Environment,**11*(3), 227–235.

[CR28] National Bureau of Statistics (NBS) [Tanzania] & ICF Macro. (2011). *Tanzania Demographic and Health Survey 2010*. Dar es Salaam, Tanzania: NBS and ICF Macro. https://dhsprogram.com/pubs/pdf/FR243/FR243[24June2011].pdf

[CR29] Onsomu, E. O., Kimani, J. K., Abuya, B. A., Arif, A. A., Moore, D., Duren-Winfield, V., & Harwell, G. (2013). Delaying sexual debut as a strategy for reducing HIV epidemic in Kenya. *African Journal of Reproductive Health,**17*(2), 46–57. 24069751

[CR31] Reda, A. A., & Lindstrom, D. (2014). Recent trends in the timing of first sex and marriage among young women in Ethiopia. *Etude De La Population Africaine= African Population Studies,**28*(2 Suppl), 1157. 27011431 10.11564/28-0-564 PMC4800999

[CR32] Ryu, D.-H. (2023). Trends in early sexual initiation and its association with socio-environmental factors among Korean adolescents. *Children,**10*(4), 613. 10.3390/children10040613 37189862 10.3390/children10040613 PMC10136636

[CR33] Saikia, R., & Barman, M. P. (2017). A review on accelerated failure time models. *International Journal of Statistics and Systems,**12*(2), 311–322.

[CR34] Shrestha, R., Karki, P., & Copenhaver, M. (2016). Early sexual debut: A risk factor for STIs/HIV acquisition among a nationally representative sample of adults in Nepal. *Journal of Community Health,**41*, 70–77. 26184108 10.1007/s10900-015-0065-6 PMC4715759

[CR35] Spouses, C. (2001). Early marriage. *Innocenti Digest, 7*. https://www.girlsnotbrides.org/documents/120/UNICEF-Early-Marrage-child-spouses-Innocenti-digest-2001.pdf

[CR36] Swindell, W. R. (2009). Accelerated failure time models provide a useful statistical framework for aging research. *Experimental Gerontology,**44*(3), 190–200. 19007875 10.1016/j.exger.2008.10.005 PMC2718836

[CR37] Upchurch, D. M., Lillard, L. A., Aneshensel, C. S., & Li, N. F. (2002). Inconsistencies in reporting the occurrence and timing of first intercourse among adolescents. *Journal of Sex Research,**39*(3), 197–206. 10.1080/00224490209552142 12476267 10.1080/00224490209552142

[CR38] Urassa, M., Marston, M., Mangya, C., Materu, J., Elsabe, D., Safari, Kh., Kagoye, S., Todd, J., & Boerma, T. (2024). Cohort profile update: Magu Health and Demographic Surveillance System, Tanzania. *International Journal of Epidemiology,**53*(3), Article dyae058. 38676640 10.1093/ije/dyae058 PMC11055399

[CR39] Wellings, K., Collumbien, M., Slaymaker, E., Singh, S., Hodges, Z., Patel, D., & Bajos, N. (2006). Sexual behaviour in context: A global perspective. *The Lancet,**368*(9548), 1706–1728. 10.1016/S0140-6736(06)69479-8 17098090

[CR40] Wringe, A., Cremin, I., Todd, J., McGrath, N., Kasamba, I., Herbst, K., Mushore, P., Żaba, B., & Slaymaker, E. (2009). Comparative assessment of the quality of age-at-event reporting in three HIV cohort studies in sub-Saharan Africa. *Sexually Transmitted Infections,**85*(Suppl_1), i56. 19307342 10.1136/sti.2008.033423 PMC2654104

[CR41] Zaba, B., Boerma, T., Pisani, E., & Baptiste, N. (2002). Estimation of levels and trends in age at first sex from surveys using survival analysis. *MEASURE Evaluation Project, Carolina Population Center Working Paper*(0251). https://www.measureevaluation.org/resources/publications/wp-02-51.html

[CR42] Zaba, B., Pisani, E., Slaymaker, E., & Boerma, J. T. (2004). Age at first sex: Understanding recent trends in African demographic surveys. *Sexually Transmitted Infections,**80*(suppl 2), ii28–ii35. 15572637 10.1136/sti.2004.012674 PMC1765851

